# Performance and Health Parameters of Sows and Their Litters Using a Probiotic Supplement Composed of *Bacillus subtilis* 541 and *Bacillus amyloliquefaciens* 516

**DOI:** 10.3390/ani14233511

**Published:** 2024-12-04

**Authors:** Aline Maria Silva Barbosa, Maria Paula Souza Carvalho, Luciana de Paula Naves, Stephane Alverina Briguente da Motta, Rhuan Filipe Chaves, Maíra Resende, Daniele de Lima, Lea Hübertz Birch Hansen, Vinícius de Souza Cantarelli

**Affiliations:** 1 Faculty of Medicine Veterinary and Animal Science, Federal University of Lavras, Lavras 37200-000, MG, Brazilluciananaves@ufla.br (L.d.P.N.); 1briguente@gmail.com (S.A.B.d.M.); 2 Animalnutri Ciência e Tecnologia, José de Santana, Centro, 520, Patos de Minas 38700-052, MG, Brazil; rhuan@animalnutri.com.br (R.F.C.); maira@animalnutri.com.br (M.R.); 3 Novonesis, Animal Biosolutions, Sales 13278-327, SP, Brazil; 4 Novonesis, Animal Biosolutions, Business Unit, 2800 Kongens Lyngby, Denmark

**Keywords:** feed additive, functional nutrition, gut health, piglets, probiotic, sows

## Abstract

This study investigated the efficacy of using probiotics on the performance and health parameters of sows and their litters. A randomized block design was used with 584 sows and 292 replications, with two dietary treatments: the control group (basal diet without probiotics) and the probiotic group (basal diet supplemented with 400 g/ton of a probiotic composed of *Bacillus subtilis* (*B. subtilis*) 541 and *Bacillus amyloliquefaciens* (*B. amyloliquefaciens*) 516). Feed intake was evaluated throughout the experimental period. Bodyweight and backfat thickness of the sows were measured at the beginning and end of each phase. Piglets were weighed individually at birth and at weaning. Performance variables and physiological parameters were analyzed. Sows that received the probiotic supplement exhibited increased milk production (*p* = 0.05) and bodyweight loss, along with reduced postpartum cortisol levels (*p* < 0.05). The piglets from the probiotic treatment group had higher (*p* < 0.001) weaning weight and fewer (*p* < 0.05) crushing deaths, received fewer (*p* < 0.001) medications, and had lower (*p* < 0.05) excretion of pathogenic bacteria and lower (*p* < 0.05) excretion of fecal *Lactobacillus* sp. They also had higher (*p* < 0.05) concentration of fecal myeloperoxidase (MPO) close to weaning and improved ileal histomorphometric measures. In conclusion, supplementation with the probiotic product improves performance and promotes health parameters of the sows their litters.

## Simple Summary

The use of probiotic supplements in pig feed is a nutritional tool that promotes gut health. Probiotics are live microorganisms that make up the gut microbiota and provide a physiological benefit to the host. In this article, we evaluate the potential of a probiotic supplement composed of *Bacillus subtilis* (*B. subtilis*) 541 and *Bacillus amyloliquefaciens* (*B. amyloliquefaciens*) 516 as a feed additive for sows in the gestation and lactation periods. We found that the probiotic treatment had a positive effect on the performance and physiological parameters of the sows and their litter.

## 1. Introduction

There are some critical points in the swine production system that make it challenging. Currently, hyperprolificacy is a key issue because it directly impacts the health and welfare of sows and the survival and quality of piglets [1]. Hyperprolific sows are more prone to dysbiosis and oxidative stress conditions, leading to cell damage [2].

Nutrition plays an important role in healthy development of the animals, increasing productivity and profitability to keep the production system viable [3]. The reproductive performance of hyperprolific sows and the growth performance of piglets are the most important aspects that affect the economic efficiency of the modern swine production industry [4].

Currently, there are various nutritional tools available to maximize nutrient utilization, directly influencing performance in swine production. Among these tools, the use of probiotics stands out, as their colonization in the intestine provides benefits for both sows and piglets [5]. Probiotics help reduce stress and inflammation [6]. Probiotics compete with pathogenic microorganisms for nutrients and adhesion sites on the intestinal mucosa, inhibiting the growth of harmful bacteria and maintaining the balance of the intestinal microbiota [7].

The microbiota and its products are indispensable not only for intestinal development but also to shape the innate immune system of the host, thus exercising multifaceted impacts on gut health [8]. In this regard, gut health plays an important role in providing immunity and greater resistance to pathogenic infections. When gut health is compromised, digestion and nutrient absorption are affected, reducing performance, leading to economic losses and greater susceptibility to diseases [9].

Bacteria of the *Bacillus* genus have been used as a nutritional tool for different phases of the swine production system. The use of *Bacillus subtilis* (*B. subtilis*) enhances the host’s welfare by preventing the proliferation of pathogens, which reduces the risk of gastrointestinal diseases and, consequently, lowers stress and discomfort in the animals. Additionally, *B. subtilis* lowers intestinal pH through acid fermentation, creating an environment that favors beneficial bacteria while inhibiting harmful microorganisms. This balanced gut environment not only improves nutrient absorption but also stimulates the immune system associated with the intestine, leading to better overall health outcomes [10,11,12,13]. In parallel, *Bacillus amyloliquefaciens* (*B. amyloliquefaciens*) complements these benefits by producing extracellular enzymes such as cellulase, α-amylases, proteases, and metalloproteases. These enzymes enhance the efficiency of nutrient digestion and absorption, further contributing to improved growth performance, reproductive efficiency, and resilience to stress. Together, these two species of *Bacillus* significantly contribute to the health and productivity of swine, highlighting their value as nutritional supplements in modern pig production [14,15,16].

Therefore, this study was conducted to investigate the probiotic product efficacy of *B. subtilis* 541 and *B. amyloliquefaciens* 516 under Brazilian farm conditions, as supplement in the feeds of sows during gestation and lactation over a reproductive cycle, with the aim of evaluating its effects on performance and health parameters of sows and suckling piglets.

## 2. Materials and Methods

### 2.1. Site and Ethics Committee

The experiment was conducted at Fazenda São Paulo, a commercial farm in the municipality/county of Oliveira, MG, Brazil. All the procedures used in this study were approved by the Ethics Committee on Animal Use of the company Animalnutri Ciência e Tecnologia, Patos de Minas, MG, Brazil (protocol n^o^.013/22).

### 2.2. Animals, Facilities, and Experimental Design

In the gestation phase, a total of 584 sows, primiparous and multiparous females up to the eighth parity, were used. They were divided into three groups with 186, 198, and 200 sows, respectively. The groups were arranged in time blocks, with a two-month interval between the first and second groups and a one-month interval between the second and third groups. The sows came from commercial hybrid lines of DB Agricultura e Pecuária (DB 90 females × LQ 1250 males) and Agroceres PIC (Camborough females × AGPIC 337 males).

In the lactation phase, 508 sows were evaluated, consisting of 106 primiparous and 402 multiparous females up to the eighth parity. Seventy-six sows were removed from the trial due to complications during the gestation period. As a result, 162 sows were evaluated in the first group, 167 in the second group, and 179 in the third group.

The pregnant sows were housed in barns consisting of individual pens (2.25 × 0.65 m) equipped with semiautomatic nipple drinkers and semiautomatic feed dispensers, where they remained up to 110 days of gestation, at which point they were transferred to farrowing facilities. A total of 21 farrowing rooms were used, seven for each group. The farrowing rooms contained semiautomatic curtains and farrowing crates (2.25 × 1.80 m) equipped with semiautomatic nipple drinkers, semiautomatic feed dispensers, and a creep area with heat lamps to warm the piglets.

The sows of each group were distributed into two treatments. The distribution of sows into two treatments was in a randomized block design, considering parity order, weight, genetics, and backfat thickness as blocks. Considering the three groups monitored, 292 sows were evaluated per treatment; each sow and litter constituted an experimental unit.

### 2.3. Experimental Procedures

For each group, the sows were monitored throughout a complete reproductive cycle and their litter throughout the suckling phase, corresponding to an experimental period of approximately 144 days. The sows of the control treatment received a basal diet without addition of the probiotic supplement, following the nutritional formulation used by the swine farm. The sows in the probiotic treatment received the basal diet supplemented *on top*, via the premix, with 400 g of the probiotic formulation per ton of feed. The commercial probiotic product evaluated is composed of *B. subtilis* 541 and *B. amyloliquefaciens* 516, with a minimum concentration of 2.75 × 10^9^ CFU/g, according to the manufacturer, Novonesis, Valinhos, São Paulo, Brazil. The excipient of the probiotic supplement is calcium carbonate.

The feeding program for the sows included three diets: gestation 1, gestation 2, and the same diet for both prepartum and lactation phases, provided during the periods of 1–28, 29–90, and 91–115 days of gestation, respectively (Table 1). The basal diets of the sows and piglets were formulated to meet or exceed nutritional requirements as defined by the NRC [17]. Each day, each sow received 2.0 kg for the gestation 1 diet, 1.8 kg for the gestation 2 diet, and 2.4 kg for the prepartum diet. Feed was supplied once a day, in the morning. Sows and piglets had free access to water throughout the experimental period.

On day 110 of gestation, the sows were transferred to the farrowing facilities. The sows of the control treatment remained in lactation for 20 ± 1.84 days and those of the probiotic treatment for 20 ± 1.74 days. Throughout the lactation period, feed was supplied ad libitum, and feeding occurred four times a day, following the management practices used by the farm. For this phase, the feed was in bags and supplied manually using scoops.

Soon after birth, the piglets were identified using numbered ear tags. Litter size was standardized through cross-fostering within the first three days of life, according to the treatments. The control treatment was maintained with 14 ± 2.23 piglets, and the probiotic treatment with 14 ± 2.59 piglets per sow. The piglets received creep feeding as of 10 days of age up to the time of weaning: for the control treatment, 20 ± 1.84 days, and for the probiotic treatment, 20 ± 1.74 days. A single creep feed (Table 2) was supplied for all the piglets from the sows of the two treatments evaluated. The health condition of the sows and piglets was monitored daily, and all medication supplied was recorded (date, medication, dosage, and reason). Reasons for removing sows from the study were lameness, metritis, abortion, prolapse, dystocia, retention of piglets during birth, and excessive weight loss due to health problems.

### 2.4. Parameters Evaluated

#### Performance of the Sows and Their Litters

All the sows and their respective piglets were considered to determine performance parameters. The feed intake of the sows was determined at the end of each phase, considering the amount of feed supplied and the leftover feed in the period. Each sow was weighed individually (Weightech^®^ scale, model NT—3000 pro) from São Paulo, SP, Brazil, at the beginning and end of gestation as well as at the end of lactation to calculate weight gain during gestation and weight gain or loss during lactation. At these same times, backfat thickness was measured at the P2 point (65 mm from the edge of the dorsal mid-line, at the level of the last rib) [18] using an ultrasound device (Xuzhou Kaixin^®^, model KX 5600) from Xuzhou, JS, China. All piglets were weighed individually at birth, and at weaning (Weightech^®^ scale, model NT—1000 LED) from São Paulo, SP, Brazil. The average daily weight gain of the piglets was calculated. At parturition of each sow, the following data were recorded: time of birth, sex of the piglets, and the number of piglets born alive, stillborn, and mummified. Piglet deaths over the lactation period were recorded along with their apparent cause. To quantify sow milk production, the following formula was used: milk production (g/day) = [(0.718 × average daily weight gain of the piglet (g) − 4.9) × number of piglets]/0.19 [19].

### 2.5. Feces Score of the Piglets During the Lactation Period

**Figure 1:**
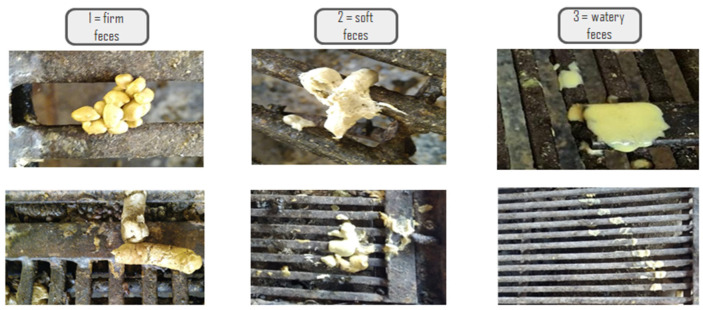
Fecal score classification.

Once a day, throughout the lactation period, the feces of per pen/litter piglets was visually observed and classified as firm (score 1), soft (score 2), or watery (score 3), according to the methodology described by [20] and as shown in Figure 1.

### 2.6. Salivary Cortisol in Sows

Ten sows from each treatment, all from the second group, were selected for saliva collection, considering their parity order, weight, genetics, and backfat thickness. The sows selected for salivary cortisol analysis were not used for collection of the other biological samples evaluated. Collection was carried out in two moments: the first collection was made on the expected day of farrowing, which, for the control treatment, occurred 2.2 days prior to the actual date of farrowing, and for the probiotic treatment, the collections were 1.3 days prior to the actual date of farrowing. The second collection was made six days after farrowing. The saliva samples were collected using cotton as a swab, attached to a string, which was manually inserted in the sows’ cheeks. The cotton remained in the sow’s mouth until approximately 2.0 mL of saliva was obtained. When saturated, the cotton swabs were placed in 5.0 mL hypodermic syringes (Descarpack^®^) from São Paulo, SP, Brazil and the plunger was pressed to extract the saliva, which was immediately placed in microtubes and stored in a freezer at −20 °C. The salivary cortisol concentration was determined at the Laboratório Imunova Análises Biológicas LTDA (Curitiba, Paraná, Brazil) using chemiluminescence analytic principles (Roche Diagnostics Laval, Laval, QC, Canada), according to the methodology of [21].

### 2.7. Immunoglobins in the Blood and Colostrum

One day after parturition, and after litter size was standardized through cross-fostering, ten sows from each treatment evaluated in the second group were selected for blood and colostrum sampling, considering parity order, weight, genetics, and backfat thickness. In addition, one piglet (female) between 4 and 18 days of age from each one of these sows was also selected for collection of blood samples, for a total of ten animals per treatment. The selection criterion was the piglet (female) with weight nearest the average weight of the litter.

The selected sows were restrained with a snare, and blood was drawn from the jugular vein using a disposable hypodermic needle (Injex^®^ 40 × 12 mm) from Ourinhos, SP, Brazil. and collected in tubes containing a clot activator. These tubes contain a spray-coated clot activator on the tube wall, which accelerates the coagulation process. After manual restraint of the piglets, blood aliquots were drawn from the jugular vein into tubes containing a clot activator using a disposable hypodermic needle (BD^®^ 13 × 0.3 mm) from Curitiba, PR, Brazil. The blood samples of the sows and piglets were centrifuged at 2000× *g* for 15 min (Daiki^®^ centrifuge, model 80-2B) from Araucária, PR, Brazil, and the serum was stored in microtubes at −20 °C until analyses were performed.

One hour after parturition, approximately 25 mL of colostrum was obtained by manual milking. The teats of the sows were cleaned with wet wipes and 70% alcohol. Thoracic, abdominal, and inguinal teats were milked to obtain a pooled sample from all the teats. The colostrum aliquots were stored in Falcon tubes (KASVI^®^) from Pinhais, PR, Brazil, at −20 °C until analyses were performed.

The concentrations of IgA, IgM, and IgG in the blood serum of the sows and piglets and in the colostrum were determined using membrane immunoassay, Western blot, and dot blot techniques, respectively, according to the methodology of [22]. The C-reactive protein levels in the serum of the piglets were determined using the immunoturbidimetric method, following the methodology of [23]. All the serum and colostrum analyses were performed at the Laboratório Imunova Análises Biológicas LTDA (Curitiba, PR, Brazil).

### 2.8. Mucosal Inflammatory Markers and Ileal Histomorphometry of the Piglets

At 18 days of age, 10 piglets from each treatment group were anesthetized by electronarcosis, followed by exsanguination through sectioning of the cervical artery and cranial vena cava. Subsequently, the abdominal cavity was opened to expose the ileum. The ileal mucosa was scraped in the luminal surface region to quantify the concentrations of the following interleukins: IL-1β, IL-4, IL-6, IL-8, IL-10, IL-12/23p40, Interferon Alpha (INF-α), Interferon Gamma (INF-γ), and the Tumor Necrosis Factor Alpha (TNF-α). The scraped content was stored in sterile microtubes and stored at −20 °C. The samples were processed following the guidelines of the commercial kit (Invitrogen TM, EPX090-60829-901) from Waltham, MA, USA, which applies the Luminexx MAP technique to determine the interleukin concentrations.

Ileal fragments of approximately 4.0 cm were collected from each piglet, specifically 10 cm anterior to the ileocecal junction, to analyze tissue samples for ileal histomorphometry. The fragments were washed with a 0.9% physiological saline solution and then fixed in a 10% formaldehyde solution for 48 h. After that period, they were washed with two 70% alcohol baths, and the fragments remained in the solution until histological processing. The samples then underwent standard histological processing, and the slides were stained with hematoxylin and eosin.

**Figure 2:**
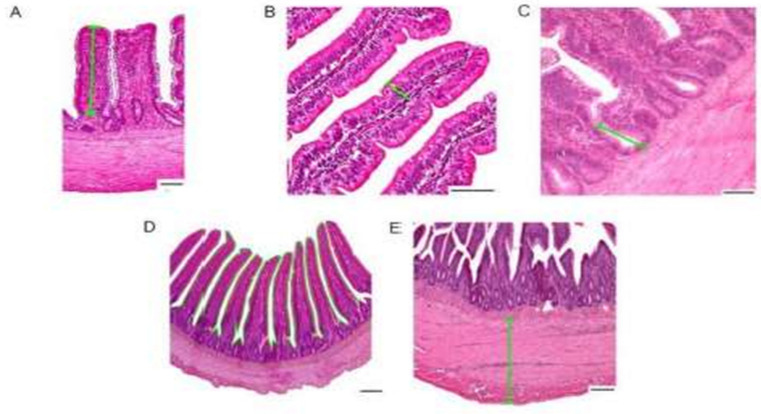
Morphometric measurements: (**A**) villus height; (**B**) epithelial height; (**C**) crypt depth; (**D**) mucosal surface enlargement factor (the continuous line represents the mucosal surface along the villus length); (**E**) muscular layer thickness. Scale bar: 100 µm for (**A**–**E**). 40× magnification. Adapted from [24].

The following parameters were measured: villus height, epithelial height, crypt depth, intestinal mucosal surface, and muscular layer/coat thickness, as shown in Figure 2. First, a panoramic image of the entire histological section was taken, followed by three images in greater magnification of this tissue. Three different points were identified in each image to measure the parameters described above. Analyses of serum interleukins and histological analyses were performed by the Imunova Análises Biológicas LTDA Laboratory (Curitiba, Paraná, Brazil).

### 2.9. Bacterial Count and Myeloperoxidase Activity in Feces

One day after parturition, approximately 5.0 g of feces from each female was collected through rectal stimulation and stored in a Falcon tube at 4 °C for maximum two days. The samples were then sent to the laboratory, where the total spore count of *Bacillus*, lactic acid bacteria, *Escherichia coli* (*E. coli*), and *Clostridium perfringens* (*C. perfringens*) was performed.

*E. coli* and *Bacillus* were counted using the spread plate technique. For coliforms, 1.0 mL of the dilutions was inoculated in duplicate for each sample (Petri dishes—90 × 150 mm), and 20 mL of Chromocult® agar Merck, from Darmstadt, HE, Alemanha, was added and incubated under aerobic conditions at 37 °C for 24 h. Then, the colonies were counted in the dilution, which contained from 25 to 250 colonies (pink colonies = non-*E. coli* coliforms and violet = *E. coli* coliforms). For the lactic bacteria, in turn, 10 µL of the dilutions was inoculated in duplicate for each sample (12-well microplates with 2.0 mL of Rogosa agar in each well) and incubated at 37 °C in an anaerobic jar with 5% to 10% CO_2_ for 72 h. Then, the colonies were counted in the dilution, which contained from 10 to 100 colonies. For *C. perfringens*, the pour plate technique was used, according to the procedure of [25]. Bacteria were counted using a colony counter, and the results were expressed in colony-forming units per gram (CFU/g).

Piglet fecal samples were collected at two points in time, at 7 and 17 days of age, pooling the feces of all the piglets of the same experimental unit. To extract feces, rectal stimulation with cotton swabs was performed on all the piglets in the pen. All the feces were stored in sterilized plastic bags, and then, the feces were manually homogenized, creating a pooled piglet fecal sample. Two samples were taken from the pool: one for analysis of bacteria count (the same bacteria previously mentioned for the sows) and another for analysis of myeloperoxidase (MPO).

To evaluate MPO, the feces were stored in 1.5 mL microtubes and frozen at −20 °C. The tetramethylbenzidine (TMB) reagent method was used for this analysis, with TMB as a substrate in the presence of a hydrogen peroxide solution, which stimulates release of MPO. The reaction was detected in the wavelength of 620 nm [26]. Microbial count and MPO activity in the feces were determined by the Centro de Diagnósticos de Sanidade Animal (Concórdia, Santa Catarina, Brazil).

## 3. Statistical Analyses

Statistical analyses were performed using the Rstudio version 4.2.1 software (R Core Team, Vienna, Austria). All the data were tested for normality using the Shapiro–Wilk and Barlett tests. Variables that did not follow normal distribution or homoscedasticity were transformed (Box–Cox/Ordernorm). The treatment effects were analyzed using mixed models with the Imer procedure (R Code Team, Viena, Austria). The group, farrowing room, genetics, and parity order were included in the model as random effects in all the analyses.

For piglets, litter weight, average daily weight gain, and days in lactation were included as fixed effects. The pregnancy rate, medication, and cause of piglet death were analyzed using the chi-square test through the Rstudio statistical package. The incidence of diarrhea was analyzed using the Imer procedure (R Core Team, Viena, Austria), and the data were adjusted using binomial distribution.

All the data are described as LSMEANS, and the standard error of the mean (SEM) for each variable is presented. The difference between mean values was considered statistically significant when *p* < 0.05 and a tendency when the *p*-value was between 0.05 and 0.10.

## 4. Results

The sows that received the probiotic product had greater (*p* < 0.05) weight loss in lactation and higher milk production (Table 3). The two groups evaluated did not differ from each other (*p* > 0.05) regarding backfat thickness, average daily feed intake, wean-to-estrus interval, farrowing rate, and length of parturition. In addition, differences (*p* > 0.05) were not observed in relation to number of piglets and piglet weight at the time of birth.

The piglets from sows that received the probiotic product in the diet had a longer lactation period (*p* < 0.001) and higher weaning weight (*p* < 0.001) (Table 4). However, the greater weight gain (*p* < 0.05) of this group remained even after necessary adjustments were made. Thus, with weight adjustment to the 21st day of age, the effect of different days of lactation between the groups was eliminated, allowing accurate comparison of the effect of the probiotic on average daily weight gain of the piglets.

Pre-weaning mortality did not differ between treatment groups (*p* > 0.05). The probiotic group had a lower number of piglet deaths due to crushing (*p* < 0.05), although a larger number of piglets were removed from the experiment (*p* < 0.05). Yet, the piglets raised by probiotic-supplemented sows received less medication (*p* < 0.001) and had a lower medication use attributed to arthritis (*p* < 0.05). The dietary treatments did not affect (*p* > 0.05) the scores and incidence of diarrhea in the piglets (Table 5).

The level of salivary cortisol in sows from the probiotic treatment was higher pre-farrowing and lower in the postpartum period compared to those in the control (*p* < 0.01) (Table 6).

The concentrations of immunoglobin A, M, and G in the colostrum and in the serum of the sows one day after parturition were not affected (*p* > 0.05) by the addition of the probiotic product to the feed, except from IgA in colostrum trending to be higher in the control treatment sows (*p* < 0.10) (Table 7). Piglets raised by sows fed the diet containing the probiotic product had a lower (*p* < 0.05) level of IgA in the serum at 18 days of age, yet there was no difference (*p* > 0.05) in the concentration of this protein at 4 days of age. On day 4, IgM levels in piglet serum tended to be higher in the probiotic treatment (*p* < 0.10). Probiotic administration did not further influence the concentration of immunoglobulins and C-reactive protein in piglets.

The fecal bacterial count of the sows one day after parturition showed similar results (*p* > 0.05) between the treatments for non-*E. coli* coliforms, *E. coli* coliforms, total coliforms, *Lactobacillus* sp., and *C. perfringens* (Table 8). The sows fed the diet containing probiotics had a higher (*p* < 0.05) fecal count of *Bacillus* sp., as was likewise observed for their progeny at 7 and 17 days of age. There was no effect (*p* > 0.05) of the dietary probiotic in relation to the count of coliforms, *Lactobacillus* sp., and *C. perfringens* or on MPO activity in the feces of the piglets at 7 days. However, in the collection of feces on day 17, the piglets raised by the sows fed diets without probiotics had a higher count (*p* < 0.05) of coliforms, *Lactobacillus* sp., and *C. perfringens* and lower (*p* < 0.05) MPO activity. The piglets of the probiotic group had lower coliforms, *Lactobacillus* sp., and *C. perfringens* and higher MPO.

**Figure 3:**
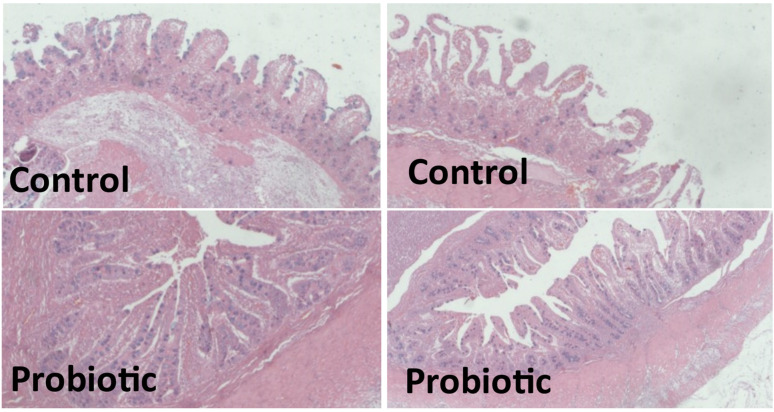
Effect of the addition of the probiotic in the diets of pregnant and lactating sows on the ileal histomorphometry of piglets at 18 days of age. Values based on a total of 20 observations.

The concentrations of the anti-inflammatory cytokines in ileal mucosa, namely IL-4 and IL-10, were similar (*p* > 0.05) across the treatments (Table 9). The concentration of IL-6 was higher (*p* < 0.05) in the piglets from the control treatment. For the other pro-inflammatory cytokines analyzed, i.e., IFN-α, IFN-γ, IL-1β, IL-8, TNF-α, and IL-12, there was no effect (*p* > 0.05) of the treatments. Supplementing the sow feeds with the probiotic did not affect (*p* > 0.05) villus height, muscular layer thickness, goblet cells, or the villus/crypt ratio in the ileal segment of the piglets. However, including the probiotic in the sow feeds resulted in higher (*p* < 0.05) values for the measurements of mucosal surface area, crypt depth, and epithelial height of the piglets (Figure 3).

## 5. Discussion

Feed additives are widely used in livestock production to enhance production efficiency. Dietary supplementation of probiotics for sows can improve the health of the offspring by affecting the gut microbiota [27]. In the present study, sows fed diets supplemented with probiotics had a higher body weight loss during lactation and higher milk production. This greater mobilization of body reserves may have been directed to milk production.

Our study corroborates the findings of [28], in which sows supplemented with the probiotic product had higher milk production. However, it contrasts with the studies of [29,30], who reported lower weight loss of sows in the lactation period when fed with *B. subtilis* C 3102 and *B. subtilis* 541 + *B. amyloliquefaciens* 516, respectively. In other studies [31,32], differences were not found in the weight of sows that received diets supplemented with probiotics based on *B. subtilis* + *Lactobacillus acidophilus* and *B. subtilis + B. licheniformis*, respectively.

Probiotics can enhance digestive enzyme activity and nutrient absorption by improving intestinal integrity and modulating the microbiota composition [33]. In addition, ref. [34] emphasized that as probiotics are associated with beneficial microbiota, they contribute to an increase in short-chain fatty acids, which is reflected in greater availability of energy for milk production.

Probiotic supplementation to sows in the gestation and lactation phases led to higher piglet weaning weight and, consequently, higher ADG. This improvement in weight gain can also be attributed to the increased availability of milk from the sow to the piglets. Additionally, the enhanced performance of the piglets is linked to the protective role of probiotics on the intestinal epithelium, where they compete with pathogenic bacteria for nutrients and absorption sites [35]. This resulted in greater mucosal surface area and epithelial height, as confirmed in our histomorphometric analyses. Therefore, there may have been greater utilization of the nutrients in the diet since there was a greater intestinal absorption area.

Analysis of the number of medications showed that the piglets of the probiotic treatment received fewer medications and had less arthritis than the piglets of the control treatment. With these data, we see the positive impact of the probiotic product on sow and piglet health. Probiotics contribute to regulate host health in various ways, improving digestion, nutrient absorption, and immune response; increasing the concentration of beneficial intestinal microorganisms; and inhibiting pathogenic bacteria, thus acting to regulate intestinal diseases [36].

Our results were like those of [32], in which the body weight and ADG of the sucking piglets increased along with supplementation of *B. subtilis* and *B. licheniformis* in the diet of the sows. Ref. [37] likewise observed higher weaning weight in piglets supplemented with *B. amyloliquefaciens*. In our study, the piglets from the probiotic treatment showed a 24% reduction in mortality due to crushing. This reduction may be related to the lower cortisol levels found in the sows that received the diet supplemented with the probiotic product.

In a similar study, ref. [5] concluded that the use of probiotics reduced cortisol levels and increased serotonin levels in sows, which in turn serves as an indicator of well-being. Lower cortisol levels may indicate a state of stress relief and improved maternal behavior in sows [38]. It is well established that elevated cortisol levels are strictly related to stress in sows, which results in stereotypical behaviors that, in turn, can increase the crushing rate of piglets. Thus, sows with higher cortisol levels tend to show less attention and care for the litter, which raises the risk of mortality due to crushing.

The result for females in the control treatment showed lower levels of salivary cortisol in the pre-farrowing period. However, this result is possibly associated with the time of collection. The first collection was made on the expected date of farrowing. Females not treated with probiotics farrowed 2.2 days after collection of saliva. In contrast, females fed with probiotics farrowed 1.3 days after saliva collection. At the second time of collection, six days post-farrowing, females that consumed probiotics showed lower levels of salivary cortisol. Percent increase in cortisol from pre- to post farrowing was 172% and 4% for the control and probiotic groups, respectively. These results indicate a positive effect of the probiotic on the female’s physiological condition, reducing cortisol levels.

An increase in cortisol in the early postpartum period may suggest that parturition has stress-inducing aspects [39]. A study conducted by [40] showed that sucking piglets from sows fed a diet supplemented with *B. subtilis* PB6 had lower cortisol levels (on day 14 and day 21), indicating relief from the stress the piglets confront during the nursing period. The authors attributed this result to the colonization of the intestinal mucosa by *Bacillus* and a consequent reduction in pathogens, leading to reduced inflammation, which then reduced stress. In a study conducted with probiotics for broilers [41], it was observed that the use of these products can mitigate the effects of thermal stress by promoting improvements in intestinal morphology and barrier function. This enhancement leads to greater resilience against infections and inflammation in the animals, which supports our results.

Variations in serum biochemical indices are the result of changes in the permeability of tissue cells, which can modify metabolic function [42]. Probiotics can improve host immunity by modulating the immune system [43]. In the current study, the piglets raised by sows supplemented with the probiotic product had a lower IgA serum concentration at 18 d. This lower systemic IgA value near weaning is likely due to the tendency for a lower concentration of this immunoglobin in the colostrum (Table 7), where the piglets of this treatment had lower intake of this protein at birth.

In the present study, the *Bacillus* count in the feces of the sows treated with probiotics was higher, as expected, since the sows of this treatment received supplementation with the *Bacillus*-based probiotic. The fecal microbiota of pigs undergoes remarkably rapid changes after birth, becoming more diverse with age [44]. The *Bacillus* strains supplemented to the sows were already detected in piglet feces at 7 days of age, even though the probiotic had not been directly consumed by the offspring. That shows vertical transfer from the sow to the piglets and clearly shows that maternal supplementation is an effective means of probiotic colonization early in piglet life. On the other hand, the levels of IgM were higher in 4-day-old piglets raised by sows fed probiotics. This finding aligns with a previous study [30], suggesting that probiotic supplementation may have played a role in modulating the immune response of the piglets, promoting the production of IgM. Furthermore, the diversification of the intestinal microbiota resulting from supplementation may have contributed to the maturation of the immune system in the animals, further enhancing the effectiveness of the immune response.

In addition, our study showed an increase in the concentration of *Bacillus* strains in the feces of piglets at 17 days. We observed that, besides maternal milk, piglets had other sources of colonization, such as contact with the sow’s feces and access to the sow’s feed trough, which could confer greater microbiota diversity and potentially enhance resilience against pathogens. The increase in *Bacillus* in the piglets’ feces on day 17 appears to be related to the reduction in counts of non-*E. coli* coliforms, *E. coli* coliforms, total coliforms, and *C. perfringens*. This effect is likely due to the probiotic’s mechanism of action, which includes competition for adhesion sites, modulation of intestinal bacterial populations, and enhancement of barrier function, thereby inhibiting pathogenic bacteria adhesion [45]. The observation of a higher number *of Lactobacillus* sp. in the control group, as opposed to the piglets treated with *B. subtilis* and *B. subtilis + B. methylotrophicus*, as seen in studies [46,47], likely reflects the competitive nature of *Bacillus*-containing probiotics within the gut microbiota. *Bacillus* probiotics can effectively colonize the intestinal tract, often outcompeting other bacteria, such as *Lactobacillus*, by monopolizing nutrients and attachment sites on the intestinal mucosa. This competitive interaction may contribute to a reduction in *Lactobacillus* populations, allowing *Bacillus* strains to establish dominance within the gut environment.

In the analysis of fecal MPO, the piglets raised by sows supplemented with the probiotic product demonstrated significantly greater enzymatic activity in their feces near weaning. The increase in MPO activity reflects the bactericidal action of neutrophils in response to the production of reactive oxygen species, resulting in the denaturation of the enzyme and the release of MPO [48]. Thus, MPO activity is classified as an inflammation marker, as it indicates both oxidative and inflammatory activity [49]. However, it is important to consider that animals are subjected to constant challenges, and an elevated MPO response in the probiotic group may be related to a faster and more efficient immune response, especially in response to the natural challenge of the farm, which manifests as diarrhea. The piglets in both treatments experienced episodes of diarrhea for more than 50% of the lactation period (Table 5).

Consequently, it is possible that the piglets in the probiotic group demonstrated a more rapid immune response, given that the two treatment groups showed similar levels of diarrhea. This expression of MPO was crucial due to the natural challenge of the farm and the presence of diarrhea in piglets during the lactation phase. MPO plays an important role in several diseases, particularly those associated with neutrophil-mediated inflammation, such as inflammatory bowel disease [50]. In such conditions, a significant increase in the activity of this enzyme is observed [51]. We associate this immune response with improved zootechnical performance and better intestinal integrity in piglets subjected to probiotic treatment.

Probiotics can maintain the intestinal microbiota balance, increase digestive capacity, improve mucosal immunity, and inhibit proliferation of pathogens in the intestine [52]. Probiotics can protect the intestine by competing with pathogens for attachment, strengthening the tight junctions between enterocytes, and increasing the mucosal immune response to pathogens. Interaction between microorganisms and epithelial cells is the beginning of the host’s immune response, which can eliminate potential pathogenic microorganisms [53].

Our study showed that the piglets raised by sows supplemented with the probiotic product had lower concentration of the pro-inflammatory IL-6 cytokine in the ileum. IL-6 plays an important role in regulating the intestinal immune response, increasing the resistance of the intestinal barrier, and activating neutrophils and IgA of B cells, which are important components in defense against enteric infections [54]. IL-6 is produced immediately and transitorily in response to infections and tissue injury and contributes to host defense by stimulating the acute phase, hematopoiesis, and immune reactions [55].

Ref. [27] also found lower levels of plasma IL-6 in piglets from a probiotic treatment based on *Lactobacillus plantarum* B90 and *Saccharomyces cerevisiae* P11. Ref. [56] likewise found reduced IL-6 in the ileal mucosa of fattened pigs treated with *B. amyloliquefaciens*. Previous studies showed different results regarding the same cytokine. Ref. [57] found greater expression of ileal IL-6 in piglets supplemented with probiotics based on *Pediococcus acidilactici* and *Saccharomyces cerevisiae boulardii*. Ref. [37], in turn, did not observe a difference in the concentration of serum IL-6 between piglets that were supplemented and not supplemented with *B. amyloliquefaciens.*

For the other cytokines, no significant values were found. However, there was a trend towards a reduction in the expression of IL-1β (pg/mg) in the group treated with probiotics, suggesting that probiotic supplementation may have contributed to an increase in the immunity of the piglets. The study by [58] highlighted that supplementation with probiotics such as *Clostridium butyricum*, *Bacillus subtilis*, and *B. licheniformis* significantly reduced serum and ileal concentrations of TNF-α and IL-1β at 28 days. Additionally, serum concentrations of IL-6 were significantly reduced on days 14 and 28.

Contrary to the findings reported by [58], we did not find a significant difference for TNF-α but only a numerical increase in animals treated with probiotics, which may be related to the lack of an adequate challenge. The absence of significant results for some variables, such as cytokines, may be attributed to the lack of a challenge in the animals. Under more challenging conditions, such as thermal or pathological stress, the immune responses may be more consistent, resulting in more significant data. This highlights the need for further research to clarify the effects of supplementation in females and their consequences on the health and performance of piglets.

The intestinal barrier and the microbiota coevolve early in life and are reciprocally related, resulting in the establishment of a mature intestinal ecosystem. Maternal probiotic supplementation can help maintain the integrity of the intestinal barrier of the offspring early in life [59]. There was no significant result to correlate dietary treatment with the villus/crypt ratio; however, there was an increase in mucosal surface area, crypt depth, and epithelial height in the ileal segment, indicating a greater absorption area and a more developed immune system. Such changes in the ileal mucosa lead promote better conditions for nutrient digestion and absorption. It also means that there is no need to expend considerable amounts of energy and nutrients to repair the intestinal mucosa. Thus, it is understood that probiotic supplementation of sows during gestation and lactation led to better gut health in the piglets, which is consistent with the better performance of the litter at weaning, such as higher body weight.

Our results are supported by [30], who evaluated the same probiotic product and observed thicker ileal mucosa in piglets from the probiotic treatment. Similar results were reported by [60] using probiotics based on *B. mesentericus* and *C. butyricum*. In a similar way, ref. [61] reported the tendency of higher villus height and ileal crypt depth in piglets from a *B. altitudinis*-based probiotic treatment. Probiotics are able to modify the histology of the intestinal mucosa. The structure of the intestinal mucosa is a crucial factor for digestive and absorptive functions, which are closely connected with growth [62].

## 6. Conclusions

Including *B. subtilis* 541 and *B. amyloliquefaciens* 516 in the diet of gestating and lactating sows increases milk production and leads to lower postpartum cortisol levels, thus improving sow welfare and performance. This supplementation also benefits the offspring, promoting greater weight gain, increased intestinal mucosal surface area, and reduced inflammatory cytokine production. Furthermore, probiotic supplementation modulates the microbiota of both sows and piglets, promoting microbial diversity, which contributes to improved gut health and immune function in piglets. These benefits support greater resistance and resilience in piglets during the weaning process. Therefore, maternal probiotic supplementation represents an effective strategy to not only improve sow welfare but also to enhance the health and development of the progeny.

## Figures and Tables

**Table 1:** Ingredients and nutritional composition of the basal diets for the sows in the gestation and lactation phases.

Ingredients (%)	Gestation 1	Gestation 2	Prepartum/Lactation
Corn (7.8% CP)	65.400	63.800	54.000
Soybean cake	17.800	17.800	
Soybean meal (46%)	11.800	13.500	30.000
Sugar			5.000
Soybean oil			4.000
Meat meal (45% CP)	2.600	2.300	4.000
Limestone	0.611	0.611	0.722
Salt	0.500	0.500	0.500
L-Lysine 78.8%	0.094	0.111	0.211
DL-Methionine 99%	0.078	0.100	0.189
L-Threonine 98%	0.078	0.094	0.200
L-Tryptophan 98%			0.017
L-Valine 96%			0.100
Mycotoxin adsorbent	0.200	0.200	0.200
Availa^®^ Sow ^1^	0.075	0.075	0.075
Mineral and vitamin premix ^2^	0.8	0.8	0.8
Calculated (cal.) and analyzed (anal.) nutritional composition, expressed in % ^3^			
Metabolizable energy (Kcal/kg) (cal.)	3012.00	3028.00	3424.00
Dry matter (cal.)	88.463	88.464	89.651
Ether extract (cal.)	3.488	3.419	7.089
Ether extract (anal.)	3.783	3.562	7.107
Crude protein (cal.)	14.238	14.823	20.178
Crude protein (anal.)	14.550	14.948	19.750
Crude fiber (cal.)	8.024	8.089	2.600
ADF (cal.)	9.262	9.343	2.912
NDF (cal.)	15.908	15.966	6.798
Ash (cal.)	4.825	4.854	5.643
Ash (anal.)	4.525	4.505	5.257
Available calcium (cal.)	0.902	0.902	1.098
Available phosphorus (cal.)	0.412	0.398	0.547
Total lysine (cal.)	0.757	0.774	1.240
Digestible lysine (cal.)	0.608	0.629	1.102
Digestible methionine+ cysteine (cal.)	0.465	0.495	0.677
Digestible threonine (cal.)	0.486	0.517	0.841
Digestible tryptophan (cal.)	0.114	0.119	0.208

^1^ Nutritional ingredient for swine, containing inorganic zinc, 62,500 mg/kg; manganese, 25,000 mg/kg; copper, 12,600 mg/kg; chromium, 500 mg/kg; selenium, 190 mg/kg. ^2^ Nitrogen, 15.23 g/kg; phytase, 70,000.00 IU/kg; carnitine, 6250.00 mg/kg; calcium nitrate, 96.28 g/kg; folic acid, 500.00 mg/kg; pantothenic acid, 3562.50 mg/kg; biotin, 56.25 mg/kg; copper, 1875.00 mg/kg; choline, 70.81 g/kg; chromium, 50.00 mg/kg; iron, 15.00 g/kg; iodine, 125.00 mg/kg; manganese, 6250.00 mg/kg; niacin, 5000.00 mg/kg; selenium, 56.25 mg/kg; vitamin A, 1,250,000.00 IU/kg; vitamin B1, 237.50 mg/kg; vitamin B12, 4375.00 mg/kg; vitamin B2, 2500.00 mg/kg; vitamin B6, 437.50 mg/kg; vitamin D3, 250,000.00 IU/kg; vitamin E, 6875.00 IU/kg; vitamin K3, 475.00 mg/kg; zinc, 16.25 g/kg. ^3^ According to the methodologies of the Compêndio Brasileiro de Alimentação Animal (2017). Analyses were conducted by the company CBO Análises Laboratoriais (Valinhos, São Paulo, Brazil).

**Table 2:** Ingredients and nutritional composition of the creep feed supplied to the sucking piglets.

Ingredient (%)	Amount
Corn (7.8% CP)	44.900
Soybean meal (46% CP)	15.700
Dehydrated plasma	5.000
Dehydrated milk	9.000
Dehydrated whey	20.000
Soybean oil	3.000
Vitamins ^1^	0.050
Microminerals ^2^	0.100
Phytase10,000 FTU	0.010
Antioxidant ^3^	0.020
Flavoring agent ^4^	0.050
Dicalcium phosphate (18.5% P)	0.428
Limestone	0.658
L-Lysine HCl 78.8%	0.444
DL-Methionine 99%	0.239
L-Threonine 98%	0.244
L-Tryptophan 98%	0.059
L-Valine 96%	0.085
Calculated (cal.) and analyzed (anal.) levels, expressed in % ^5^	
Metabolizable energy (Kcal/kg) (cal.)	3459.520
Ether extract (cal.)	5.649
Ether extract (anal.)	4.890
Crude protein (cal.)	20.680
Crude protein (anal.)	20.970
Digestible lysine (cal.)	1.520
Digestible methionine (cal.)	0.520
Digestible methionine + cysteine (cal.)	0.850
Digestible threonine (cal.)	1.020
Digestible tryptophan (cal.)	0.290
Digestible arginine (cal.)	1.010
Digestible valine (cal.)	1.050
Digestible isoleucine (cal.)	0.780
Digestible leucine (cal.)	1.680
Digestible histidine (cal.)	0.520
Digestible phenylalanine (cal.)	0.890
Crude fiber (cal.)	1.530
ADF (cal.)	2.737
NDF (cal.)	8.705
Ash (cal.)	3.006
Ash (anal.)	7.270
Lactose (cal.)	17.600
Total calcium (cal.)	0.880
Available phosphorus (cal.)	0.550

^1^ Vitamin A, 25,000.00 IU/kg; vitamin B1, 4.50 mg/kg; vitamin B12, 75.00 mg/kg; vitamin B2, 12.50 mg/kg; vitamin B6, 7.50 mg/kg; vitamin D, 35,000.00 IU/kg; vitamin E, 75.00 IU/kg; vitamin K, 37.50 mg/kg. ^2^ Copper, 500.00 mg/kg; iron, 250.00 mg/kg; iodine, 3.00 mg/kg; sodium, 7500.00 mg/kg; manganese, 50.00 mg/kg; zinc, 7500.00 mg/kg; selenium, 0.88 mg/kg. ^3^ Butylated hydroxytoluene (B.H.T) 375.00 mg/kg. ^4^ Herbal extracts (cinnamon, lime, cloves, orange, and grape). ^5^ According to methodologies of the Compêndio Brasileiro de Alimentação Animal (2017). Analyses performed by the company CBO Análises Laboratoriais (Valinhos, São Paulo, Brazil).

**Table 3:** Performance of sows fed the probiotic product during gestation and lactation.

Variable	Treatment		SEM	CV (%)	*p*-Value
	Control	Probiotic			
Initial body weight (kg)	231.5	232.7	12.26	17.59	0.506
Pre-partum weight (kg)	272.2	272.4	7.14	11.11	0.912
Weight at weaning (kg)	254.1	252.0	9.40	14.53	0.365
Weight loss during lactation (kg)	18.7	22.7	5.47	94.32	0.032
Initial BT (mm)	13.8	13.8	1.08	23.09	0.906
Pre-partum BT (mm)	14.7	14.3	0.84	22.30	0.164
BT at weaning (mm)	14.1	14.0	1.12	22.53	0.685
ADFI in lactation (kg)	6.9	6.8	0.43	19.63	0.705
Milk production (kg/d)	7.7	8.4	0.34	30.89	0.005
WEI (d)	5.1	4.8	0.66	53.83	0.764
Farrowing rate (%)	85.2	87.2	-	-	0.533
Length of parturition (h)	3.8	3.8	0.18	34.05	0.301
Total born (n)	17.6	17.9	0.26	15.80	0.240
Stillbirths (%)	6.0	6.3	0.30	106.98	0.509
Mummified (%)	2.4	2.3	0.28	173.97	0.811
Live born (n)	15.9	16.1	0.31	19.35	0.426
Birth weight (kg)	1.2	1.3	0.02	16.05	0.570
Mummified weight (kg)	0.2	0.2	0.02	81.13	0.219
Stillborn weight (kg)	1.0	1.0	0.03	36.78	0.597
Live-born weight (kg)	1.3	1.3	0.02	15.41	0.988
CV%, birth	24.2	24.6	0.73	32.02	0.399

Control treatment—basal diet, without probiotics; Probiotic treatment—basal diet supplemented with the probiotic product containing 2.75 × 10^9^ CFU/g *B. subtilis* 541 and *B. amyloliquefaciens* 516. Performance parameters evaluated based on a total of 508 observations. Abbreviations: SEM, standard error of mean; BT, backfat thickness; ADFI, average daily feed intake; WEI, wean-to-estrus interval; coefficient of variation. The mean values were separated by the F-test.

**Table 4:** Impact of the probiotic product that supplemented the gestation and lactation feeds on piglet performance.

Variable	Treatment		SEM	CV (%)	*p*-Value
	Control	Probiotic			
Litter size after CF (n)	14.489	14.651	0.277	14.982	0.398
Litter size at weaning (n)	12.199	12.441	0.238	14.299	0.101
PW after CF (kg)	1.328	1.333	18.074	18.074	0.801
SD after CF	0.226	0.236	0.009	32.737	0.088
CV% after CF	17.175	17.962	0.621	34.416	0.137
Weaning weight (kg)	4.812	5.143	0.123	21.474	<0.001
SD at weaning	0.993	1.068	0.038	32.214	0.025
CV% at weaning	21.254	20.93	0.759	28.744	0.643
WW—adjusted to 21 d (kg)	5.032	5.29	0.145	20.734	0.008
Days of suckling (n)	20.046	20.567	0.491	9.242	<0.001
ADG—adjusted to 21 d	0.176	0.188	0.006	25.672	0.007
ADG ^1^	0.174	0.186	0.006	25.668	0.007
Mortality and removal (%)	14.25	14.936	0.943	75.676	0.724

Control treatment—basal diet, without probiotics; Probiotic treatment—basal diet supplemented with the probiotic product containing 2.75 × 10^9^ CFU/g *B. subtilis* 541 and *B. amyloliquefaciens* 516. Values of litters corresponding to a total of 508 sows. ^1^ Using PW after CF as covariate. Abbreviations: SEM, standard error of mean; SD, standard deviation; CV, coefficient of variation; CF, cross-fostering; PW, piglet weight; WW, weaning weight; ADG, average daily weight gain; d, days; mean values were separated using the F-test.

**Table 5:** Effect of the probiotic product that supplemented the diets of gestation and lactating sows on piglet mortality, removal, medication, as well as diarrhea of piglets and sows.

Treatment	Control	Probiotic	SEM	*p*-Value
Mortality and removal				
Total deaths (n)	556	561	-	0.832
Diarrhea (%)	11.3	10.4	-	0.528
Crushed (%)	23.7	18.0	-	0.018
Debilitated ^1^ (%)	5.7	4.3	-	0.258
Removed ^2^ (%)	59.2	67.4	-	0.004
Medication ^3^ (reason)				
Total medication (n)	6381	6004	-	<0.001
Diarrhea (%)	23.4	24.0	-	0.467
Arthritis (%)	2.8	2.1	-	0.009
Diarrhea in piglets				
Score 1 (%)	48.0	48.0	3.67	0.967
Score 2 (%)	35.9	35.8	3.00	0.877
Score 3 (%)	14.1	14.3	4.49	0.788
Mediated score	1.7	1.7	0.10	0.922
Days with diarrhea (%)	52.0	52.0	3.67	0.967

Control treatment—basal diet, without probiotics; Probiotic treatment—basal diet supplemented with the probiotic product containing 2.75 × 10^9^ CFU/g *B. subtilis* 541 and *B. amyloliquefaciens* 516. ^1^ Locomotor and/or injury problems. ^2^ Piglets not in uniformity with the litter and removed as part of cross-fostering to standardize litter size. ^3^ Colistin^®^; Excenel^®^; Gentamox^®^; Pencivet^®^. Abbreviations: SEM, standard error of mean. The means were separated by the F-test.

**Table 6:** Effect of the probiotic product supplemented to gestating and lactating sows on the concentration of cortisol in sows.

Variable	Treatment		SEM	CV (%)	*p*-Value
	Control	Probiotic			
Pre-farrowing salivar cortisol (µg/dL)	0.408	0.577	0.247	92.23	0.001
Postpartum salivar cortisol (µg/dL)	1.110	0.600	0.502	136.66	<0.001

Control treatment—basal diet, without probiotics; Probiotic treatment—basal diet supplemented with the probiotic product containing 2.75 × 10^9^ CFU/g *B. subtilis* 541 and *B. amyloliquefaciens* 516. Values based on a total of 20 observations. Pre-farrowing saliva cortisol collection T1 = 2.2 days and T2 = 1.3 days prior to actual farrowing. Postpartum saliva cortisol collection T1 = 6 days and T2 = 6 days. Abbreviations: SEM, standard error of mean; CV, coefficient of variation. The mean values were separated by the F-test.

**Table 7:** Effect of the probiotic product that supplemented the diets of pregnant and lactating sows on the concentrations of immunoglobins in colostrum and serum of sows and piglets as well as C-reactive protein in piglets.

Variables	Treatment		SEM	CV (%)	*p*-Value
	Control	Probiotic			
Immunoglobins of sows					
IgA (mg/dL) in colostrum	0.999	0.816	0.101	27.157	0.099
IgM (mg/dL) in colostrum	0.911	0.943	0.102	24.309	0.773
IgG (mg/dL) in colostrum	1.000	0.912	0.134	29.465	0.620
IgA (mg/dL) in serum—1 day after parturition	0.986	0.830	1.153	35.638	0.267
IgM (mg/dL) in serum—1 day after parturition	0.998	0.780	0.171	44.802	0.253
IgG (mg/dL) in serum—1 day after parturition	1.000	0.922	0.170	28.102	0.664
Immunoglobins of piglets					
IgA (mg/dL) in serum—4 day	1.058	0.988	0.159	29.660	0.652
IgM (mg/dL) in serum—4 day	0.936	1.278	0.211	40.254	0.081
IgG (mg/dL) in serum—4 day	1.022	1.135	0.192	30.972	0.437
C-reactive protein (mg/L)—4 day	2.350	2.520	0.969	80.358	0.893
IgA (mg/dL)in serum—18 day	1.010	0.703	0.128	36.731	0.003
IgM (mg/dL) in serum—18 day	0.995	1.108	0.116	22.427	0.262
IgG (mg/dL) in serum—18 day	1.030	0.969	0.093	21.904	0.416
C-reactive protein (mg/L)—18 day	1.349	1.428	0.311	45.470	0.802

Control treatment—basal diet, without probiotics; Probiotic treatment—basal diet supplemented with the probiotic product containing 2.75 × 10^9^ CFU/g *B. subtilis* 541 and *B. amyloliquefaciens* 516. Values based on a total of 20 observations for sows and 20 for piglets. Abbreviations: SEM, standard error of mean; CV, coefficient of variation; IgA, immunoglobin A; IgM, immunoglobin M; IgG, immunoglobin G. The means were separated by the F-test.

**Table 8:** Effect of the probiotic product that supplemented the diets of pregnant and lactating sows on bacterial count and MPO in the feces of sows and piglets.

Variables	Treatment		SEM	CV (%)	*p*-Value
	Control	Probiotic			
Sows—1 day after parturition					
Non-*E. coli* coliforms	8.22	8.41	7.786	69.995	0.127
*E. coli* coliforms	7.38	7.53	6.950	72.112	0.294
Total coliforms	8.30	8.37	7.856	80.233	0.806
*Bacillus* sp.	4.20	6.89	6.839	418.577	<0.001
*Lactobacillus* sp.	8.20	8.23	7.787	86.203	0.954
*Clostridium perfringens*	5.76	6.68	6.330	196.397	0.173
Piglets—7 days of life					
Non-*E. coli* coliforms	7.49	7.47	7.076	94.713	0.935
*E. coli* coliforms	8.18	8.20	7.551	55.657	0.750
Total coliforms	8.25	8.33	7.768	70.852	0.810
*Bacillus* sp.	2.65	4.04	3.677	215.295	<0.001
*Lactobacillus* sp.	8.42	8.36	7.820	60.144	0.540
*Clostridium perfringens*	8.37	8.47	8.212	148.544	0.363
MPO (min. gtissue^−1^)	38.18	31.78	5.452	43.186	0.357
Piglets—17 days of life					
Non-*E. coli* coliforms	9.99	9.28	9.643	69.995	0.003
*E. coli* coliforms	9.28	8.72	8.672	105.904	0.003
Total coliforms	9.45	8.86	8.934	112.288	0.005
*Bacillus* sp.	3.11	4.91	5.398	176.293	<0.001
*Lactobacillus* sp.	8.41	8.11	7.839	78.097	0.003
*Clostridium perfringens*	7.30	6.71	7.398	336.759	0.040
MPO (min. gtissue^−1^)	33.58	61.27	12.544	51.391	0.006

Control treatment—basal diet, without probiotics; bacterial count data were log_10_ transformed. Probiotic treatment—basal diet supplemented with the probiotic product containing 2.75 × 109 CFU/g *B. subtilis* 541 and *B. amyloliquefaciens* 516. Values based on a total of 20 observations for sows and 20 for piglets. Abbreviations: SEM, standard error of mean; CV, coefficient of variation; MPO, myeloperoxidase. The means were separated by the F-test.

**Table 9:** Effect of the probiotic product that supplemented the diets of pregnant and lactating sows on the concentration of cytokines in ileal mucosa and ileal histomorphometry of piglets at 18 days of age.

Variables	Treatment		SEM	CV (%)	*p*-Value
	Control	Probiotic			
Cytokines of the ileal mucosa					
IFN-α (pg/mg)	1.749	1.756	0.176	13.681	0.753
IFN-γ (pg/mg)	19.107	14.863	5.554	69.052	0.520
IL-10 (pg/mg)	87.467	82.909	20.835	34.173	0.760
IL-1β (pg/mg)	574.725	390.456	119.946	50.782	0.101
IL-4 (pg/mg)	5.264	6.696	1.386	40.105	0.151
IL-6 (pg/mg)	87.740	34.027	28.043	70.792	0.025
IL-8 (pg/mg)	3831.930	4982.340	983.068	53.675	0.288
TNF-α (pg/mg)	29.521	34.353	6.567	44.382	0.379
IL-12/IL-23p40 (pg/mg)	150.341	154.619	10.851	15.124	0.715
Ileal histomorphometry					
Mucosal surface (µm)	65,326.60	91,202.00	4574.85	21.913	<0.001
Villus height (µm)	287.131	296.815	23.212	15.959	0.605
Crypt depth (µm)	57.435	65.502	5.100	20.024	0.036
Muscular layer (µm)	313.878	283.103	36.065	28.760	0.422
Goblet cells (cell/villus)	19.563	17.834	1.895	17.639	0.197
Epithelial height (µm)	20.898	25.200	1.389	14.718	<0.001
Villus/crypt ratio	5.110	4.660	0.434	22.083	0.237

Control treatment—basal diet, without probiotics; Probiotic treatment—basal diet supplemented with the probiotic product containing 2.75 × 10^9^ CFU/g *B. subtilis* 541 and *B. amyloliquefaciens* 516. Values based on a total of 20 observations. Abbreviations: SEM, standard error of mean; CV, coefficient of variation; IFN-α, interferon alpha; IFN-γ, interferon gamma, IL-10, interleukin 10; IL-1β, interleukin 1 beta; IL-4, interleukin 4; IL-6, interleukin 6; IL-8, interleukin 8; TNF-α, tumor necrosis factor alpha; IL-12/IL-23p40, interleukin 12/interleukin 23p40. The means were separated by the F-test.

## Data Availability

Data are contained within the article.
